# Game-Theoretic Motion Planning with Perception Uncertainty and Right-of-Way Constraints

**DOI:** 10.3390/s24248177

**Published:** 2024-12-21

**Authors:** Pouya Panahandeh, Ahmad Reza Alghooneh, Mohammad Pirani, Baris Fidan, Amir Khajepour

**Affiliations:** 1 Mechanical and Mechatronics Engineering Department, University of Waterloo, 200 University Ave W, Waterloo, ON N2L 3G1, Canada; aralghooneh@uwaterloo.ca (A.R.A.); fidan@uwaterloo.ca (B.F.); a.khajepour@uwaterloo.ca (A.K.); 2 Department of Mechanical Engineering, University of Ottawa, Ottawa, ON K1N 6N5, Canada; mpirani@uottawa.ca

**Keywords:** motion planning, autonomous vehicle, uncertainty, sensor fusion, game theory

## Abstract

This paper addresses two challenges in AV motion planning: adherence to right-of-way and handling uncertainties, using two game-theoretic frameworks, namely Stackelberg and Nash Bayesian (Bayesian). By modeling the interactions between road users as a hierarchical relationship, the proposed approach enables the AV to strategically optimize its trajectory while considering the actions and priorities of other road users. Additionally, the Bayesian equilibrium aspect of the framework incorporates probabilistic beliefs and updates them based on sensor measurements, allowing the AV to make informed decisions in the presence of uncertainty in the sensory system. Experimental assessments demonstrate the efficacy of the approach, emphasizing its ability to improve the reliability and adaptability of AV motion planning.

## 1. Introduction

Motion planning plays a crucial role in enabling safe and efficient navigation for AVs [1,2,3]. However, in real-world scenarios, uncertainties arise from inherent limitations in sensor measurements [4,5], imperfect knowledge of the environment, and the presence of unpredictable factors such as pedestrian movements or other vehicles’ behavior. Ignoring uncertainty can lead to conservative or overly aggressive decision-making, which can compromise safety and efficiency. Furthermore, ensuring proper adherence to right-of-way [6,7,8], a fundamental principle of traffic rules becomes essential for the safe integration of AVs into mixed traffic environments. Building on previous research, various methods have been proposed to tackle motion-planning challenges. In [9], Graph Neural Networks (GNNs) are used to predict edge feasibility in roadmaps, significantly reducing the need for computationally expensive collision checks. By learning the structure of feasible connections in the configuration space from prior data, this approach accelerates the planning process while maintaining high solution quality. In [10], the application of an improved Hopfield neural network for path planning is explored to address the limitations of the traditional Hopfield model. The proposed approach integrates the A* algorithm to preselect favorable nodes in the search area, which are then transformed into neurons for the neural network. By leveraging the A* algorithm’s ability to efficiently narrow the search space and the Hopfield network’s stability for optimization, the method achieves significant improvements in path planning. In [11], a path-planning technique for mobile robots navigating obstacle-filled environments is presented, using Cellular Neural Networks (CNNs) for image processing. When the distance to the final target is long, intermediate targets are introduced to enhance navigation. The algorithm relies on accurately identifying the start and target locations in the captured image. A major challenge is wave propagation, where the processing time varies with the distance between the start and target, and intermediate targets are selected when the wavefront does not reach the target. In [12], the complex 3D path-planning problem for Unmanned Aerial Vehicles (UAVs) is addressed, considering multiple constraints such as safety, economy, and flyability. The proposed algorithm enhances the Brain Storm Optimization (BSO) method by incorporating crossover recombination from differential evolution to improve both search capability and robustness. In [13], an approach is presented to ensure safety in the motion planning of AVs using reinforcement learning (RL) with stability guarantees. This method integrates risk-identification techniques and Lyapunov functions with the Soft Actor-Critic (SAC) algorithm. The Lyapunov function guarantees that the vehicle’s trajectory remains within a safe region, even with limited hardware resources, while the risk-sensitive learning algorithm balances reward maximization and risk minimization. In [14], a novel end-to-end learnable network is introduced for AVs, integrating perception, prediction, and motion planning into a unified framework. This approach stands out using a semantic occupancy representation, which is both interpretable and differentiable, as part of the motion-planning cost. This ensures that the planning process aligns with the vehicle’s perception and prediction estimates. The network is trained end-to-end from human driving demonstrations, enabling it to learn behaviors that are both human-like and safer. In [15], a neural network-based method is introduced to efficiently plan safe paths in uncertain, nonconvex environments. Unlike traditional approaches that depend on convex approximations and Gaussian assumptions, this framework leverages probabilistic point cloud maps and neural networks to quickly generate near-optimal paths while maintaining an acceptable level of collision risk. In [16], the Firefly Algorithm (FA), a type of swarm intelligence, is applied to mobile robot navigation in uncertain and dynamic environments. The algorithm mimics the natural attraction between fireflies based on their brightness variations, which enhances the robot’s ability to navigate, explore, and avoid obstacles efficiently. The research in [17] addresses environmental uncertainties in path planning by employing bio-heat-based modeling for needle insertion in soft tissues. It focuses on deformation-induced uncertainties, using finite element modeling to optimize trajectories by integrating temperature distributions with tissue deformation. This approach facilitates precise obstacle avoidance and provides real-time feedback during the planning process. Current research discusses the challenges associated with motion planning under perception uncertainty and right-of-way constraints for AVs and addresses them using game theory [18,19,20].

Stackelberg equilibrium [21,22,23], a fundamental concept in game theory, addresses strategic interactions in hierarchical decision-making settings where a leader’s actions shape follower responses. This equilibrium finds widespread applications in AV control and navigation. In [24], the multi-agent optimal tracking problem is addressed. In [25], An implementation of a traffic simulation utilizing a 3-person Stackelberg game is introduced to replicate human behavior in driving situations. The simulation assumes that each vehicle is engaged in a 3-person game, where the given vehicle serves as the initial game leader. In [26,27], a simulator is designed to evaluate different policies in autonomous driving decision-making, including the Stackelberg approach. In [28], the lane-merging maneuver is investigated. By monitoring the speed variations of the interacting vehicle, the AV decides, based on Stackelberg equilibrium, to merge with the main traffic. In [29,30], a unified framework that encompasses decision-making and motion planning is proposed for lane-change maneuvers of AV considering the social behaviors of surrounding traffic occupants. The Stackelberg game is utilized to address the decision-making aspect, and the potential field method and Model Predictive Control (MPC) are used in motion planning. In [31], a Stackelberg routing strategy is developed for AVs in a mixed-autonomy traffic network. In [32,33], the interaction between the human driver and the vehicle in the shared steering control problem is solved by adopting the Stackelberg formulation. The primary focus of the literature lies in applying the Stackelberg solution to handle the decision-making process at a higher level for AVs. A goal addressed in this paper is to utilize this approach to tackle the motion-planning problem associated with AVs in a dynamic traffic scenario, specifically addressing the issue of the right-of-way.

Building upon Nash equilibrium [34], the Bayesian equilibrium [35,36] introduces a framework that incorporates Bayesian reasoning and uncertainty into strategic decision-making settings. This powerful extension allows players to not only consider their own strategies and payoffs but also account for probabilistic beliefs about the actions and payoffs of other players. In [37], a Bayesian game is employed to model the decision-making process of human drivers who have partial information about their surroundings. In [38], a method for making lane-change decisions, which utilizes the Bayesian game approach, is proposed with a specific emphasis on effectively handling the intense interactions that occur in dense highway traffic. In [39,40], an emergency maneuver scenario involving two AVs interacting with a road obstacle characterized by a random behavior is solved using a Bayesian game. In [41], the Risk-Constrained Robust Stochastic Bayesian Game (RC-RSBG) is developed to address a novel safety objective for interactive planning with uncertainty about other traffic participants’ behavior. In [42,43], the scenario of a pedestrian crossing in front of an AV is solved using Bayesian Gaussian Process analysis. In current research, Bayesian equilibrium is employed as a solution to address motion-planning difficulties in the presence of uncertainty in the perception system.

The key contributions of this paper are as follows:

- Incorporating the right-of-way concept into the motion-planning problem using Stackelberg equilibrium.
- Addressing perception uncertainties using Bayesian equilibrium, which incorporates the recorded movements of interacting road users.

The organization of the paper is as follows. In Section 2, the problem formulation and background related to motion planning in the context of rational decision-makers interacting is introduced. In Section 3, the framework for solving the motion-planning problem through the utilization of Stackelberg and Bayesian equilibrium concepts is explained. In Section 4, the numerical analysis and simulations are presented. In Section 5, experimental results are discussed. Finally, in Section 6, the study concludes with a summary of the main findings and conclusions.

## 2. Problem Formulation and Background

Consider a typical traffic scenario where *N* road users, denoted by i=1,2,⋯,N, interact with each other. The 2D position of road user *i* at time step *t* can be represented as $p_{i}\left(t\right)={\left[x_{i}\left(t\right)\;y_{i}\left(t\right)\right]}^{T}\in R^{2}$. Here, $x_{i}\left(t\right)$ and $y_{i}\left(t\right)$ represent the respective coordinates of the road user’s position in the Cartesian plane. Additionally, the orientation and velocity of road user *i* are denoted as $\varphi_{i}\left(t\right)$ and $v_{i}\left(t\right)$, respectively. Utilizing this information about each road user, the Stackelberg game is initially established, and the Bayesian game is subsequently constructed.

**Definition** **1.** *A Stackelberg Game is a game with complete information that consists of:*

- *Road users involved in the traffic scenario i∈{1,2,⋯,N}.*
- *Finite action set for each road user, $P_{i}=\left[p_{i}^{1},p_{i}^{2},\cdots ,p_{i}^{s_{i}}\right]$, where $s_{i}$ represents the number of actions for road user i.*
- *Utility function for each road user, $u_{i}\left(p_{i},p_{-i}\right)$, where $p_{i}$ represents the strategy chosen by road user i and $p_{-i}$ stands for the strategies chosen by all other road users except road user i.*

**Figure 1:**
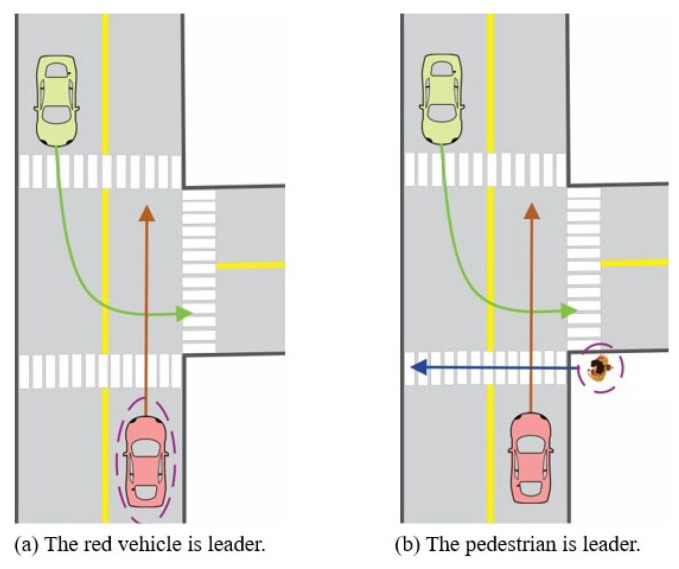
Intersection scenario.

The Stackelberg game has two types of players: leader and follower. In the interactive traffic scenario around the AV, the leader is assigned to the road user with the highest priority (right-of-way), and the rest of the road users are assigned as followers. For example, two scenarios at the intersection are displayed in Figure 1, and the road user with the highest priority is identified. In the scenario on the left, the vehicle intending to proceed straight has the highest priority, as the vehicle going straight is continuing its path without changing direction, while the vehicle turning left must cross oncoming traffic. On the other hand, in the scenario on the right, the pedestrian is afforded the highest priority, as the pedestrian is the most sensitive road user in the scenario. Within the motion-planning problem, the task is to identify the Stackelberg equilibrium involving all road users engaged in the traffic scenario. Subsequently, the actions derived from the equilibrium for the AV are regarded as the planned motion. Based on the Stackelberg game, the leader makes decisions first, taking into account the followers’ best response, and then the followers observe the leader’s decision and choose their action to form the equilibrium.

In a *N*-road user traffic scenario, let $p_{l}$ denote the action for the leader, $p_{i}$ the action for the follower *i* and $p_{-i-l}$ presents the action for other followers. The best response of follower *i* to the action of the leader and the rest of the followers is defined as,

(1) $$\begin{array}{c} B_{i}\left(p_{l},p_{-i-l}\right)=\underset{p_{i}\in P_{i}}{arg min}u_{i}\left(p_{i},p_{l},p_{-i-l}\right). \end{array}$$

A leader strategy $p_{l}^{*}$ and follower *i* strategy $p_{i}^{*}$ are in Stackelberg equilibrium if the following condition holds [25,44],

(2) $$\begin{array}{c} p_{l}^{*}=\arg\min u_{l}\left(p_{l},p_{1},\cdots ,p_{i},\cdots ,p_{N-1}\right),\\ \begin{array}{c} p_{l}\in P_{l}\\ p_{1}\in B_{1}\left(p_{l},p_{-1-l}\right)\\ \vdots \\ p_{i}\in B_{i}\left(p_{l},p_{-i-l}\right)\\ \vdots \\ p_{N-1}\in B_{N-1}\left(p_{l},p_{-(N-1)-l}\right) \end{array}\; \end{array}$$

and for the follower *i*,

(3) $$p_{i}^{*}\in B_{i}\left(p_{l}^{*},p_{-i-l}^{*}\right).$$

By satisfying the same condition as in Equation (3) for all followers, the optimal strategies for all road users are determined as $P^{*}=\left[p_{l}^{*},p_{1}^{*},\cdots ,p_{i}^{*},\cdots ,p_{N-1}^{*}\right]$ and the one that is related to AV form the motion-planning solution. The subsequent step involves defining the Bayesian game.

**Definition** **2.** *A Bayesian Game is a game with incomplete information that consists of:*

- *Road users involved in the traffic scenario i∈{1,2,⋯,N}.*
- *Finite type set for each road user, $\Theta_{i}=\left[\theta_{i}^{1},\theta_{i}^{2},\cdots ,\theta_{i}^{\eta_{i}}\right]$, where $\eta_{i}$ presents the number of types for road user i.*
- *A probability distribution over type $\theta_{i}$ as $\pi (\theta_{i})$.*
- *Finite action set for each road user $p_{i}\left(\theta_{i}\right)\in P_{i}$. It is important to recognize that each road user bases their action on their individual type without considering the types of their opponents, as they cannot observe their opponents’ types.*
- *Utility function for each road user, $u_{i}\left(p_{i}\left(\theta_{i}\right),p_{-i}\left(\theta_{-i}\right);\theta_{i},\theta_{-i}\right)$, where $\theta_{i}$ stands for the type of road user i and $\theta_{-i}$ conveys types of all other road users.*

It is important to note that traffic scenarios can be modeled as incomplete information games because not all participants have full knowledge about the types of others. In this research, it is assumed that only the AV can record the movements of other vehicles and use these data to construct a type distribution. Once the Bayesian game is established, equilibrium is searched for to find the optimal set of actions for the AV as the solution to the motion-planning problem. A Bayesian equilibrium is a situation where players, taking into account their beliefs and uncertainties about the game, make choices that are optimal given their information, and no player can improve their own expected payoff by changing their strategy unilaterally.

A strategy profile $P^{*}=\left[p_{1}^{*}\left(\theta_{1}\right),\cdots ,p_{i}^{*}\left(\theta_{i}\right),\cdots ,p_{N}^{*}\left(\theta_{N}\right)\right]$ is a Bayesian equilibrium of a game of incomplete information if the following condition holds [45],

(4) $$\begin{array}{c} \sum_{\theta_{-i}\in \Theta_{-i}}\pi \left(\theta_{-i}|\theta_{i}\right)u_{i}\left(p_{i}^{*}\left(\theta_{i}\right),p_{-i}^{*}\left(\theta_{-i}\right);\theta_{i},\theta_{-i}\right)\leq \sum_{\theta_{-i}\in \Theta_{-i}}\pi \left(\theta_{-i}|\theta_{i}\right)u_{i}\left(p_{i}\left(\theta_{i}\right),p_{-i}^{*}\left(\theta_{-i}\right);\theta_{i},\theta_{-i}\right), \end{array}$$

for every $p_{i}\left(\theta_{i}\right)\in P_{i}$, every $\theta_{i}\in \Theta_{i}$, and every road user *i*. $\pi (\theta_{-i}|\theta_{i})$ represents the probability that road user *i* assigns, after observing that his type is $\theta_{i}$, to his opponents’ types being $\theta_{-i}$. It is worth mentioning that in interactive traffic scenarios the types road user *i* detects for opponents, $\theta_{-i}$, are independent of his type, $\theta_{i}$, therefore the probability distribution $\pi (\theta_{-i}|\theta_{i})$ is simplified as $\pi (\theta_{-i})$.

## 3. Method

This section explores the fundamental requirements for establishing a game, focusing on defining strategies, utility, and belief functions for road users within the traffic scenario.

### 3.1. Strategy Function Formation

**Figure 2:**
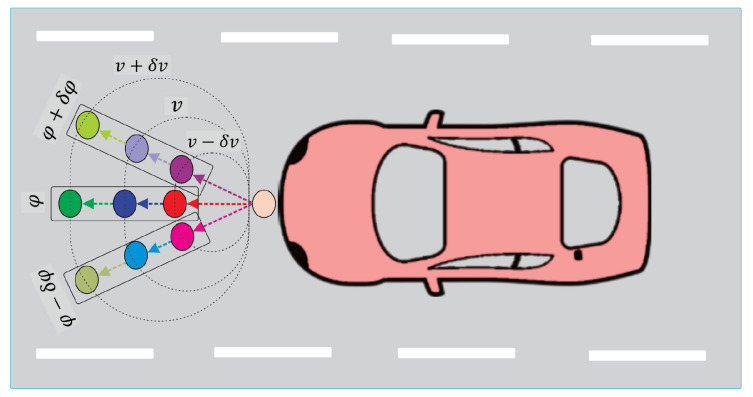
Possible nodes of road user *i* with $b_{i}:1,2,\cdots ,9$.

In the game environment, every road user adjusts their configuration by utilizing a displacement vector. It is essential to identify two key characteristics associated with this vector: its length and orientation. The length of the displacement vector determines the magnitude of the change in the road user’s configuration. On the other hand, the orientation of the displacement vector defines the direction in which the road user’s configuration is altered. Using this displacement vector, the strategy function related to each road user is identified in Algorithm 1. In this algorithm, mod(e,m) is defined as the integer modulo function that returns *e* modulo *m*, i.e., the remainder after the division of *e* by *m*, and quo(e,m) is defined as the integer quotient function that divides *e* by *m* and returns the quotient. δφ and δv are fixed parameters that define the minimum allowed modification in the direction and velocity of road users. $K_{v}$ is a parameter used for adjusting the relation between the displacement vector length and the velocity of the road user *i*. In Algorithm 1, by using the value of $b_{i}=1$ for road user *i*, a future position is established that maintains the current orientation of road user *i*, $\varphi_{i}\left(t\right)$, with a length of $K_{v}v_{i}\left(t\right)$. For $b_{i}=2$ and $b_{i}=3$, the orientation is still $\varphi_{i}\left(t\right)$; however, the length of displacement vector will be $K_{v}\left(v_{i}\left(t\right)+\delta v\right)$ and $K_{v}\left(v_{i}\left(t\right)-\delta v\right)$, respectively. For values of $b_{i}=4,5,6$, the orientation of future positions is $\varphi_{i}\left(t\right)-\delta \varphi$ and the length of displacement vector will be $K_{v}v_{i}\left(t\right)$, $K_{v}\left(v_{i}\left(t\right)+\delta v\right)$ and $K_{v}\left(v_{i}\left(t\right)-\delta v\right)$, respectively. In Figure 2, an exhibition of the future nodes of road user *i* with $s_{i}=9$ is shown.
**Algorithm 1** Strategy function$1:\;\;\;b_{i}:1,2,\cdots ,s_{i}$.$2:\;\;\;q=\mathrm{quo}(b_{i}-1,3)$   $3:\;\;\;\Delta \varphi_{i}^{b_{i}}\left(t\right)=\left\{\begin{array}{cc} \varphi_{i}\left(t\right)+\frac{q}{2}\delta \varphi \;, & \mathrm{if}\;\mathrm{mod}\left(q,2\right)=0\\ \varphi_{i}\left(t\right)-\frac{q+1}{2}\delta \varphi \;, & \mathrm{if}\;\mathrm{mod}(q,2)=1 \end{array}\right.$   $4:\;\;\;\Delta v_{i}^{b_{i}}\left(t\right)=\left\{\begin{array}{cc} K_{v}v_{i}\left(t\right), & \mathrm{if}\;\mathrm{mod}\left(b_{i},3\right)=1\\ K_{v}\left(v_{i}\left(t\right)+\delta v\right), & \mathrm{if}\;\mathrm{mod}\left(b_{i},3\right)=2\\ K_{v}\left(v_{i}\left(t\right)-\delta v\right), & \mathrm{if}\;\mathrm{mod}(b_{i},3)=0 \end{array}\right.$   $5:\;\;\;p_{i}^{b_{i}}\left(t+1\right)=\Delta v_{i}^{b_{i}}\left(t\right){\left[cos\left(\Delta \varphi_{i}^{b_{i}}\left(t\right)\right),sin\left(\Delta \varphi_{i}^{b_{i}}\left(t\right)\right)\right]}^{T}+p_{i}\left(t\right)$

### 3.2. Utility Function Calculation

In the next step, the utility function is defined for each road user. In a general traffic scenario, it is expected that the AV and other road users aim to be close to their reference path. *L* penalizes distancing from the reference path, and a shorter distance from the reference path results in a lower cost. Considering $R_{i}$ as the reference path of road user *i*, $D_{p_{1},p_{2}}$ to be the distance between points $p_{1}$ and $p_{2}$, and proj(p,R) to be the orthogonal projection of point *p* to the path R, the *L* calculation is summarized in Algorithm 2 for road user *i* who tries to leave its current node, $p_{i}\left(t\right)$, and moves to the next vertex, $p_{i}\left(t+1\right)$, where it has multiple options to select, $P_{i}\left(t+1\right)=\left[p_{i}^{1}\left(t+1\right),p_{i}^{2}\left(t+1\right),\cdots ,p_{i}^{s_{i}}\left(t+1\right)\right]$. To calculate *L* in Algorithm 2, the distance between the future node and its projection on the reference path is first measured, Dpibi(t+1),pibi′(t+1). This distance is then scaled by the distance between the current node and its projection onto the reference path, $D_{p_{i}\left(t\right),p_{i}^{\prime }\left(t\right)}$. $K_{L}$ serves as a parameter for adjusting the weight of the *L* cost.
**Algorithm 2** *L* calculationFor $b_{i}:1,2,\cdots ,s_{i}$. Find: pibi′(t+1)=proj(pibi(t+1),Ri). Find: $p_{i}^{\prime }\left(t\right)=\mathrm{proj}\left(p_{i}\left(t\right),R_{i}\right)$. Li(pibi(t+1))=KLDpi(t),pi′(t)Dpibi(t+1),pibi′(t+1).

**Figure 3:**
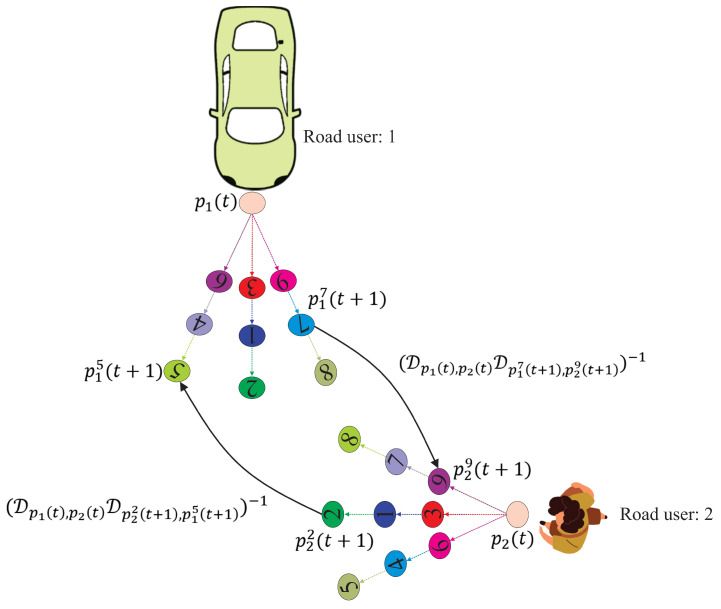
*C* calculation of vehicle-pedestrian interaction.

To avoid collision between road users, *C* is defined to manage the distance between traffic scenario participants. *C* corresponds to the distance between the future nodes of road users and is formed in a way that a longer distance results in a lower cost. Considering a general case where road user *i* wants to change its position from $p_{i}\left(t\right)$ to $p_{i}\left(t+1\right)$ in a *N*-road user interactive traffic scenario, *C* is calculated as,

(5) $$\begin{array}{c} C_{i}\left(p_{1}^{b_{1}}\left(t+1\right),\cdots ,p_{i}^{b_{i}}\left(t+1\right),\cdots ,p_{N}^{b_{N}}\left(t+1\right)\right)=K_{C}{\left(\sum_{l=1}^{N}D_{p_{i}\left(t\right),p_{l}\left(t\right)}D_{p_{i}^{b_{i}}\left(t+1\right),p_{l}^{b_{l}}\left(t+1\right)}\right)}^{-1}.\\ i:1,2,\cdots ,N\;\mathrm{and}\;b_{i}:1,2,\cdots ,s_{i}.\; \end{array}$$

where $K_{C}$ is a parameter for modifying the weight of the *C* cost. In Figure 3, *C* is calculated in an interactive traffic scenario between a vehicle and a pedestrian. Each road user has 9 nodes to choose as their future position. As shown in Figure 3, it is evident that the distance between $p_{1}^{8}\left(t+1\right)$ and $p_{2}^{8}\left(t+1\right)$ is the shortest among all possible selections of the first and second road users. Therefore, this selection will result in the highest *C* cost. Two *C* costs, $C_{1}\left(p_{1}^{7}\left(t+1\right),p_{2}^{9}\left(t+1\right)\right)$ and $C_{2}\left(p_{2}^{2}\left(t+1\right),p_{1}^{5}\left(t+1\right)\right)$, are also calculated and represented in Figure 3.

Within the dynamic traffic environment, as the expenses linked to reaching a desired destination are evaluated, another utility function known as *G* is introduced. The objective here is to evaluate how efficiently an AV or any other road user can navigate from their current position to a predefined goal point while considering the surrounding traffic conditions. Considering $\alpha_{i}$ as the intended goal of road user *i*, the *G* cost is calculated as,

(6) $$\begin{array}{c} G_{i}\left(p_{i}^{b_{i}}\left(t+1\right)\right)=K_{G}D_{p_{i}\left(t\right),\alpha_{i}}D_{p_{i}^{b_{i}}\left(t+1\right),\alpha_{i}}\\ b_{i}:1,2,\cdots ,s_{i}.\; \end{array}$$

where $K_{G}$ serves as a parameter for adjusting the weight of the *G* utility function. Next, ensuring a steady velocity with minimal alterations in a traffic environment can substantially boost both the efficiency and safety of road travel. When road users can uphold a consistent velocity, it promotes greater predictability in traffic dynamics, reducing the chances of abrupt deceleration or acceleration. Consequently, this promotes smoother traffic flow, diminishes congestion, and lowers the overall accident risk. To address this phenomenon, the *D* utility function is introduced within the traffic scenario. This cost quantifies the impact of altering road user *i* velocity as,

(7) $$\begin{array}{c} D_{i}\left(p_{i}^{b_{i}}\left(t+1\right)\right)=\left\{\begin{array}{cc} 0, & \mathrm{if}\;\mathrm{mod}(b_{i},3)=1\\ K_{D}, & \mathrm{else}\; \end{array}\right.\\ b_{i}:1,2,\ldots ,s_{i}.\; \end{array}$$

This utility function imposes a cost on future nodes whenever there is a change in velocity. Therefore, if a road user increases or decreases their velocity, $K_{D}$ will be added to their utility function. Finally, once all utility functions are computed, the final objectives corresponding to the future nodes of road user *i* in an *N*-road user interactive traffic scenario are derived by summing up *L*, *C*, *G*, and *D* as,

(8) $$\begin{array}{c} u_{i}\left(p_{1}^{b_{1}}\left(t+1\right),\cdots ,p_{i}^{b_{i}}\left(t+1\right),\cdots ,p_{N}^{b_{N}}\left(t+1\right)\right)=L_{i}\left(p_{i}^{b_{i}}\left(t+1\right)\right)+C_{i}\left(p_{1}^{b_{1}}\left(t+1\right),\cdots ,p_{i}^{b_{i}}\left(t+1\right),\cdots ,p_{N}^{b_{N}}\left(t+1\right)\right)\\ +G_{i}\left(p_{i}^{b_{i}}\left(t+1\right)\right)+D_{i}\left(p_{i}^{b_{i}}\left(t+1\right)\right).\;\\ i:1,2,\cdots ,N\;\mathrm{and}\;b_{i}:1,2,\cdots ,s_{i}.\; \end{array}$$

### 3.3. Belief Probability Function Identification

Within an interactive traffic scenario, the concept of belief encompasses the historical knowledge that each road user possesses regarding the behavior of fellow road users. Based on this information, each road user may claim a certain behavior or attitude for other participants of the traffic scenario. In the motion-planning problem, the assumption is that the AV is the sole road user capable of tracking others and holding beliefs about their behavior. In the interactive traffic scenario, road user *i* is classified into three types: normal, aggressive, and cautious. The normal type, denoted as $\theta_{i}^{1}=\theta_{i}^{n}$, represents a road user who maintains their velocity, *v*. The aggressive type, $\theta_{i}^{2}=\theta_{i}^{a}$, corresponds to a road user who increases their velocity, indicated as v+δv. Lastly, the cautious type, $\theta_{i}^{3}=\theta_{i}^{c}$, describes a road user who decreases their velocity, represented as v−δv. Following the above definition, every set of three configurations outlined in Algorithm 1, characterized by a fixed orientation, collectively constitute a single node featuring three distinct behaviors. As an example, for $b_{i}=1,2,3$, which have similar orientation in Algorithm 1, $\rho_{i}^{1}\left(\theta_{i}^{n}\right)$ is assigned as $p_{i}^{1}$, $\rho_{i}^{1}\left(\theta_{i}^{a}\right)$ as $p_{i}^{2}$, and $\rho_{i}^{1}\left(\theta_{i}^{c}\right)$ as $p_{i}^{3}$.

Next, the probability function of each type from the perspective of the AV must be determined. AV usually employs sensor measurements and object tracking algorithm [46,47] to detect and monitor other road users. A tool often employed in object tracking is the Recursive Gaussian Bayes Estimator (RGBE). One of the implementations of RGBE is the Kalman filter [48]. To track the movement of other road users, the Kalman filter often leverages the constant acceleration model [49], simplifying the process by assuming each road user maintains steady acceleration over time. In the interactive traffic scenario, individual road users being tracked are each paired with a distinct Kalman filter. This filter optimally estimates the road user *i* state, $X\left(t\right)={\left[x\left(t\right),\;y\left(t\right),\;\dot{x}\left(t\right),\;\dot{y}\left(t\right)\right]}^{T}$, by processing a sequence of measurements observed over time, Z(t). The velocity is of particular concern when considering the estimated states obtained through the Kalman filter. By gathering sufficient measurements from fellow road users and taking into account the Central Limit Theorem [50], a normal distribution is established for the measured velocity of individual road users. Considering the mean, μ, and standard deviation, σ, of the recorded velocity, the Probability Density Function (PDF) of the normal distribution is defined as,

(9) $$\begin{array}{c} f\left(v\left(t\right)\right)=\frac{1}{\left(\sigma \sqrt{2\pi }\right)}exp\left(-\frac{1}{2}{\left(\frac{v\left(t\right)-\mu }{\sigma }\right)}^{2}\right). \end{array}$$

**Figure 4:**
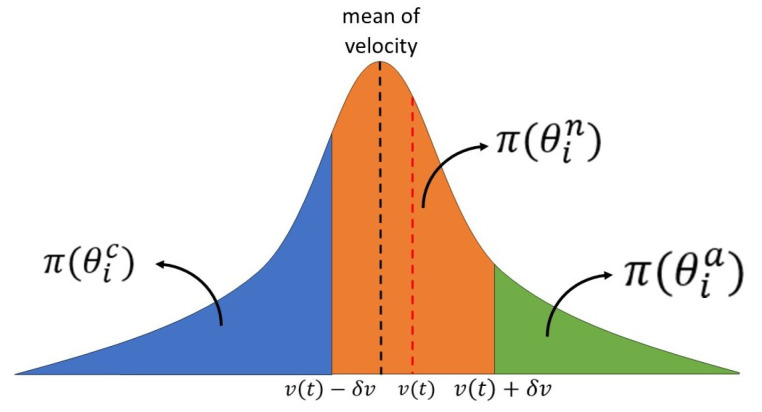
Probability distribution function.

To determine the probabilities associated with each type, an integration of the PDF for velocity over a specified range is performed. For normal behavior, the interval is defined as [v(t)−δv,v(t)+δv]. In the case of aggressive behavior, this range extends to [v(t)+δv,+∞], while for cautious behavior, it spans from [−∞, v(t)−δv]. These ranges are assigned considering the strategy function developed on Algorithm 1. This comprehensive approach allows us to capture the probabilities associated with each distinct behavioral type. An example is shown in Figure 4 to explore these ranges. The orange area denotes the probability linked to normal behavior, the blue area corresponds to cautious behavior, and the green area indicates aggressive behavior. It is important to note that for the three probabilities, the following condition holds:

(10) $$\pi \left(\theta_{i}^{n}\right)+\pi \left(\theta_{i}^{a}\right)+\pi \left(\theta_{i}^{c}\right)=1.$$

## 4. Numerical Analysis and Simulation

### 4.1. Numerical Analysis

**Figure 5:**
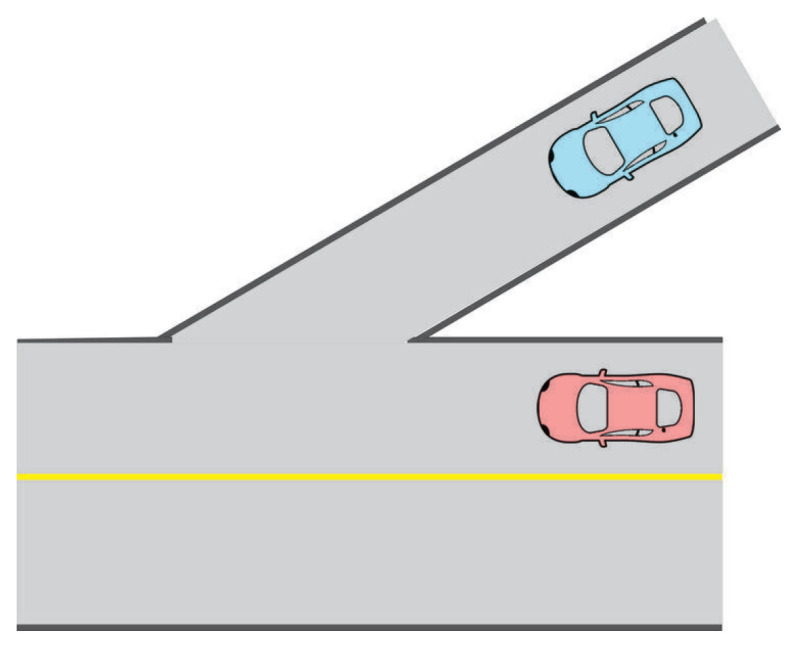
Merging scenario. Road user 1: Blue vehicle (AV). Road user 2: Red vehicle.

In this part, a scenario is examined, as shown in Figure 5, to illustrate the process of identifying equilibrium points first in a Stackelberg game and then in a Bayesian game. In this scenario, the blue vehicle (AV), road user 1, wants to merge with the main traffic and needs to interact with the red vehicle (human-driven), road user 2. In the context of the Stackelberg game, the initial step involves determining the right-of-way in the traffic scenario. Adhering to traffic regulations, the vehicle intending to merge must yield to other vehicles. As a result, the red vehicle is granted the right-of-way, allowing us to designate it as the leader, while the blue vehicle takes on the role of the follower. Using Algorithm 1, future strategies for each vehicle are identified, and using Equation (8), the associated objective functions for all strategies are calculated. When both $s_{1}$ and $s_{2}$ are configured as 6, each road user possesses a total of 6 options for selection at every step. Consequently, the bimatrix game presented in Table 1 is established accordingly, where the first entry in each cell represents $u_{2}$ and the second value shows $u_{1}$.

**Figure 6:**
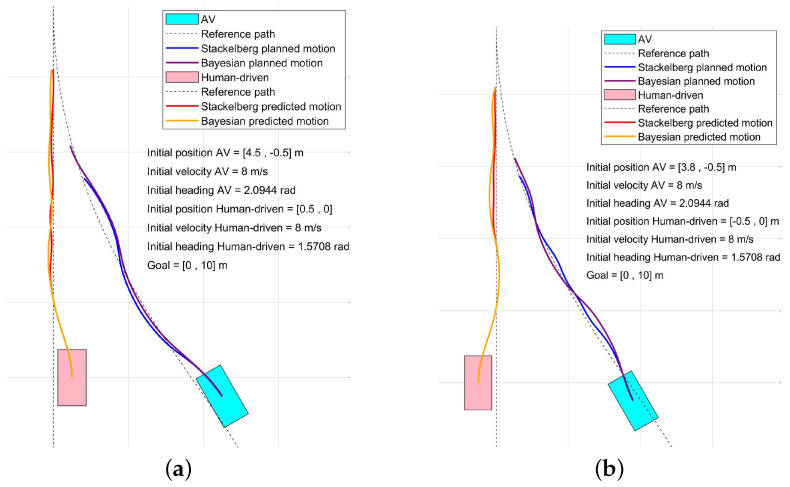
Simulation of a Merging Scenario: Comparison of Stackelberg and Bayesian motion planners. (**a**) First set of initial conditions for AV and human−driven vehicles. (**b**) The second set of initial conditions for AV and human−driven vehicles.

The best response of AV to the action of the leader is demonstrated by the red color in Table 1. Among the best responses, the leader’s optimal choice is identified as $p_{2}^{*}=p_{2}^{5}$, and correspondingly, the follower’s selection is indicated as $p_{1}^{*}=p_{1}^{5}$. Following the same procedure for each step, the trajectory related to AV is derived. In Figure 6, the planned motion for AV and predicted motion for the human-driven vehicle using the Stackelberg game are presented for two different initial conditions, spanning up to a specified prediction horizon. The game specifications are also detailed in Table 2. It is important to note that while this traffic scenario involves two road users, the same approach can be applied in situations with additional road users.

Next, the same merging scenario in Figure 5 is considered, and the Bayesian game is formulated. AV measures the velocity of the human-driven vehicle, and subsequently, it derives the normal distribution for the human-driven vehicle. Given μ=38 kph and σ=4 derived from recorded measured velocities of the human-driven vehicle, with δv=2 kph and the current velocity of the human-driven vehicle at 39.7 kph, the probability functions corresponding to various behaviors of the human-driven vehicle are as follows: $\pi (\theta_{2}^{n})=0.36$, $\pi (\theta_{2}^{a})=0.17$, and $\pi (\theta_{2}^{c})=0.47$. Using Algorithm 1 and Equation (8), the utility functions for the AV and the human-driven vehicle are computed, accounting for various human-driven vehicle behaviors. The utility calculations are documented in Table 3, Table 4 and Table 5 for normal, aggressive, and cautious behavior of human-driven vehicles, respectively, in a single step. Comparing the Stackelberg bimatrix in Table 1 with the normal behavior bimatrix in Table 3 reveals that the first and fourth rows of Table 1 correspond to the first and second rows of Table 3, respectively. Additionally, the second and fifth rows of Table 1 correspond to the first and second rows of the aggressive behavior bimatrix in Table 4. Lastly, the third and sixth rows of Table 1 correspond to the first and second rows of the cautious behavior bimatrix in Table 5. Considering the calculated probability of each type and using Equation (4), the expanded bimatrix related to the Bayesian game is represented in Table 6. The Bayesian equilibrium is identified with red color in Table 6 as well. $p_{1}^{*}=p_{1}^{1}$ forms the optimal action for AV within Bayesian equilibrium. By applying a similar methodology, all forthcoming configurations of the AV across the prediction horizon can be determined. To anticipate the trajectory of the human-driven vehicle, it is assumed that the human-driven vehicle lacks information about the AV’s behavior. Under this assumption, the same problem is solved for the human-driven vehicle by considering the AV’s various actions with equal probabilities. The result related to the interaction of AV and the human-driven vehicle in the merging scenario is shown in Figure 6 using the Bayesian game.

When comparing the outcomes of the Stackelberg and Bayesian games, it becomes evident that in the Stackelberg game, a lower speed for AV is taken. This conclusion is drawn from the fact that the planned path in the Stackelberg game is shorter than that in the Bayesian game. Stackelberg’s solution decreases the AV velocity to give right-of-way to the human-driven vehicle. On the other hand, when accounting for the probabilities associated with each possible behavior of the human-driven vehicle, the AV assumes with greater probability that the human driver will act either cautiously or normally due to higher probabilities. This probabilistic assessment enables the AV to devise a trajectory that incorporates higher speeds (covering longer distances), therefore optimizing its motion based on these predictions. Additionally, the influence of these probabilities means that the AV will occasionally choose different directions at certain points when planning its future path. This allows the AV to adapt dynamically to anticipated human behaviors. Furthermore, in the Bayesian game, where both vehicles engage in competition to merge to the main road, as is expected, the human-driven vehicle will exhibit a greater divergence from the reference path to avoid AV. It is important to note that Bayesian equilibrium may not always yield a solution, while Stackelberg equilibrium consistently does for a two-player finite game [34]. By employing a Stackelberg motion planner, it can be ensured that the motion-planning problem always has a solution.

### 4.2. Simulation

**Figure 7:**
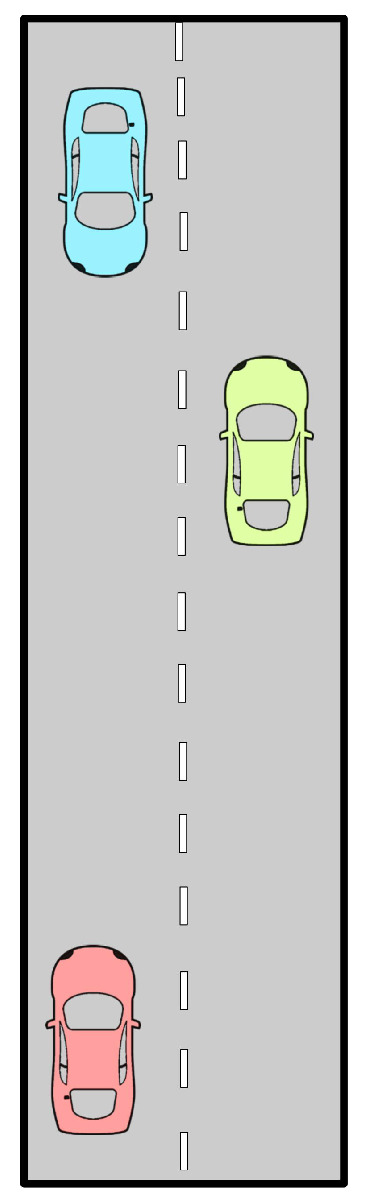
Overtaking scenario.

**Figure 8:**
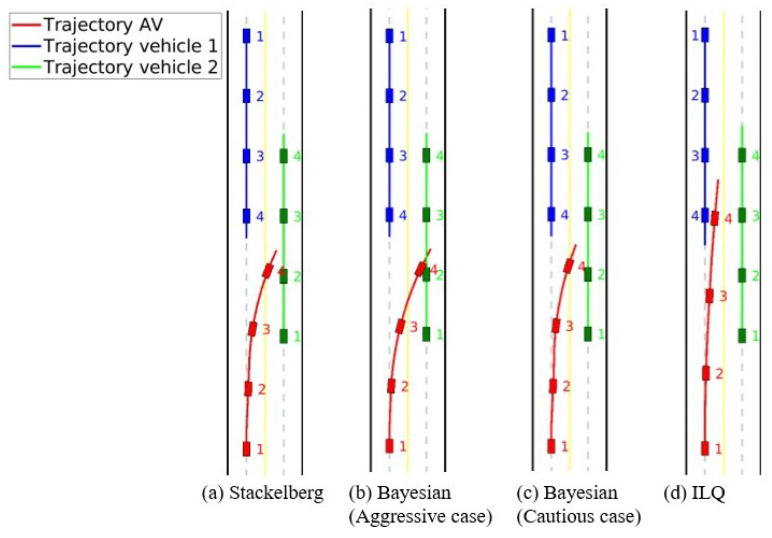
Overtaking scenario: Trajectory of road users.

**Figure 9:**
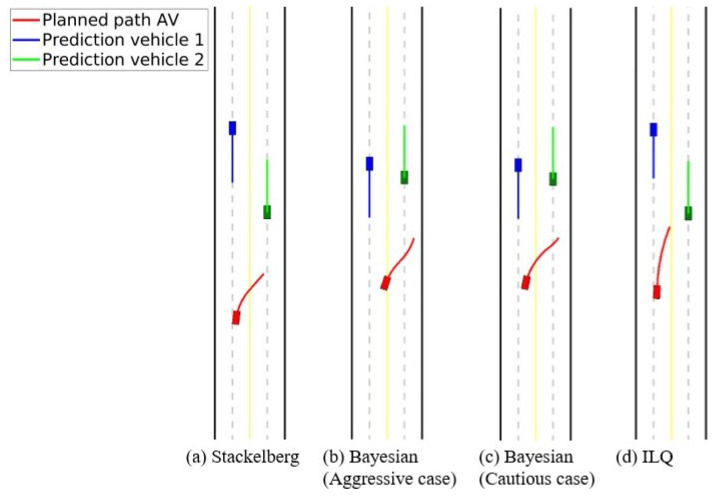
Overtaking scenario: Instance of interactions between road users.

In this part, an overtaking scenario is presented, visually depicted in Figure 7. The scenario involves an AV, represented as a red car, attempting to overtake a slower green vehicle traveling in the same direction. During this maneuver, as the AV shifts into the opposite lane to pass, it detects an oncoming blue vehicle. To prevent a potential collision, the AV must abort its overtaking attempt and merge back into its original lane. The AV’s behavior is examined under two motion-planning approaches: Stackelberg and Bayesian (with aggressive and cautious driving behaviors). For the Bayesian motion planner, when the AV encounters an oncoming vehicle perceived as aggressive (blue car), the behavior probabilities are set as follows: $\pi \left(\theta_{i}^{n}\right)=0.1,\;\pi \left(\theta_{i}^{a}\right)=0.8,\;\pi \left(\theta_{i}^{c}\right)=0.1.$ This configuration indicates a high likelihood (80%) that the oncoming vehicle will behave aggressively, suggesting that it may not slow down or yield space. The probabilities for normal and cautious behaviors are kept low at 10% each. Conversely, when the oncoming vehicle is perceived as cautious, the parameters are adjusted to: $\pi \left(\theta_{i}^{n}\right)=0.1,\;\pi \left(\theta_{i}^{a}\right)=0.1,\;\pi \left(\theta_{i}^{c}\right)=0.8.$ Here, the high probability (80%) assigned to cautious behavior implies that the oncoming vehicle is more likely to reduce speed or allow space, facilitating the AV’s overtaking maneuver. The likelihood for normal and aggressive behaviors is kept low at 10% each. For a comprehensive comparison, an additional motion-planning method based on the Iterative Linear Quadratic (ILQ) approach, as proposed in [51], is also evaluated in this scenario. The authors of [51] introduced a technique for solving general-sum differential games in which multiple agents pursue independent objectives. Utilizing feedback linearization, this method converts nonlinear system dynamics into a linear form, enabling iterative quadratic approximations to simplify the problem. The results and the trajectories of the involved road users are presented in Figure 8 and Figure 9, which correspond to the Stackelberg motion planner, Bayesian motion planner with aggressive opponent, Bayesian motion planner with cautious opponent, and the ILQ motion planner, respectively. In the Stackelberg motion planner, the oncoming blue vehicle is treated as the leader, while the AV and the slower green vehicle act as followers, adjusting their actions in response to the leader’s decisions. This hierarchical leader-follower structure enables the AV to negotiate right-of-way effectively, prioritizing safety in its maneuvering decisions. The Bayesian motion planner evaluates the AV’s response to varying levels of aggressiveness in the oncoming vehicle, highlighting how these behavioral differences affect the AV’s trajectory and motion planning.

**Figure 10:**
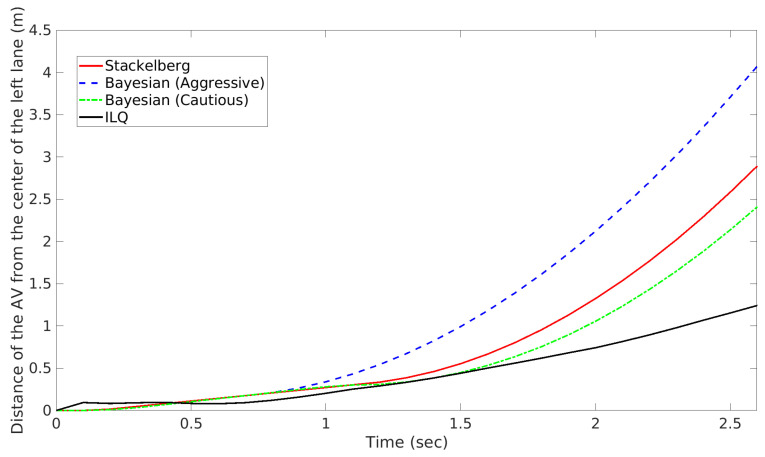
Overtaking scenario: Distance from the center of the left lane.

To thoroughly compare the planners’ performance, Figure 10 shows the distance of the AV from the center of the left lane over time. In all cases, approximately 0.5 s after the scenario begins, the planners prompt the AV to shift from the left lane back to the right lane: For the Bayesian motion planner with an aggressive opponent, the AV makes a significant and rapid deviation from the left lane, anticipating that the oncoming vehicle may act aggressively and pose a collision risk sooner. For the Bayesian motion planner with a cautious opponent, the AV makes a more gradual deviation, as it expects the oncoming vehicle to provide more space, allowing the AV to remain in the left lane longer. The Stackelberg motion planner’s performance lies between these two extremes, as it accounts for the priority of the oncoming vehicle while balancing safety and efficiency. Conversely, the ILQ motion planner demonstrates a delayed response to the oncoming blue vehicle, which could result in a collision with the oncoming blue vehicle. Additionally, another critical aspect is the computational time required for each planner to generate a complete trajectory. The entire reaction of AV in the overtaking scenario lasts approximately 2.8 s. The computation times for each planner, summarized in Table 7, reveal that while the ILQ motion planner takes about 6.14 seconds to compute, making it unsuitable for real-time application, the Stackelberg and Bayesian planners have significantly lower computation times, making them viable options for real-time motion planning.

## 5. Experimental Testing

**Figure 11:**
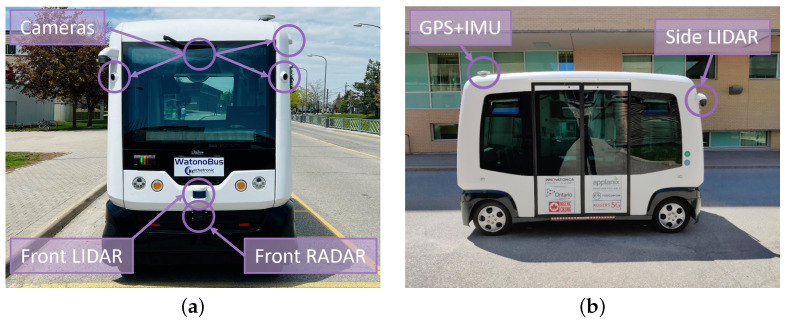
WATonoBus. (**a**) Front view. (**b**) Side view.

In this section, a detailed explanation of the experimental setup, referred to as WATonoBus, is presented. Following this, the experimental findings associated with both Stackelberg and Bayesian motion planners are provided. The WATonoBus is an AV equipped with a set of sensors, as illustrated in Figure 11, to deliver services within interactive, diverse traffic environments to gain practical experimentation and the opportunity to tackle various challenges associated with the utilization of AVs in urban areas.

The sensor suite consists of several 3.2 MP Basler Ace cameras (Basler Vision Technology, Beijing, China), three 32-channel Robosense LiDARs (Suteng Innovation Technology, Shenzhen, China), an Applanix POS LVX GNSS unit (Trimble Incorporated, Toronto, ON, Canada), and a Continental ARS408-21 RADAR (Continental AG, Hanover, Germany), all forming the vehicle’s perception system. Each sensor is essential in providing critical data to help the vehicle make informed decisions and operate safely and efficiently. The core software modules that process and interpret sensor data run on the NVIDIA Jetson Orin AGX embedded unit, which handles real-time complex computations. Additionally, visualization and control-planning modules run on an Intel NUC Ruby, offering a user-friendly interface. The Applanix GPS combines Global Navigation Satellite System (GNSS) data with Inertial Measurement Unit (IMU) data to deliver precise positioning and orientation for WATonoBus, forming the localization module. All these software modules communicate and share information through the ROS framework, facilitating smooth information flow and effective coordination between the various components of the WATonoBus system.

**Figure 12:**
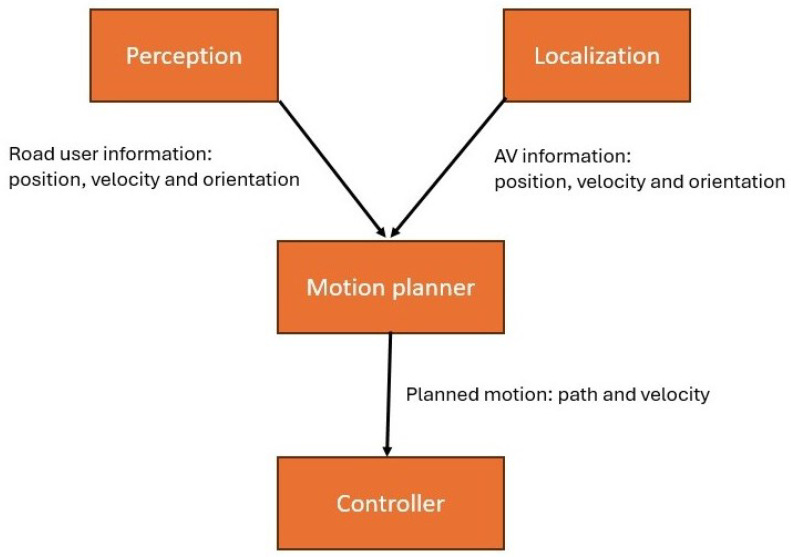
System integration of WATonoBus.

The University of Waterloo’s Ring Road, a 2.7 km curvy road, is selected as the testing environment. A frequent occurrence on Ring Road is the presence of pedestrian crossings. To assess and draw a comparison between the efficacy of the Stackelberg and Bayesian motion planners, an experimental investigation was carried out within the context of a pedestrian crossing scenario. The data pertaining to the pedestrian’s location, orientation, and velocity is gathered through a fusion of sensors inside the perception module, Figure 12. The localization module provides information regarding the position, orientation, and velocity of WATonoBus, Figure 12. This information serves as the foundational input for the construction of both the Stackelberg and Bayesian game models. In the subsequent phase, the results obtained from these game-theoretic motion planners are transmitted to the lateral and longitudinal control systems, Figure 12. In the context of lateral control, an MPC controller is employed, and for longitudinal control, a feedforward controller is deployed, offering effective velocity adjustment for WATonoBus.

**Figure 13:**
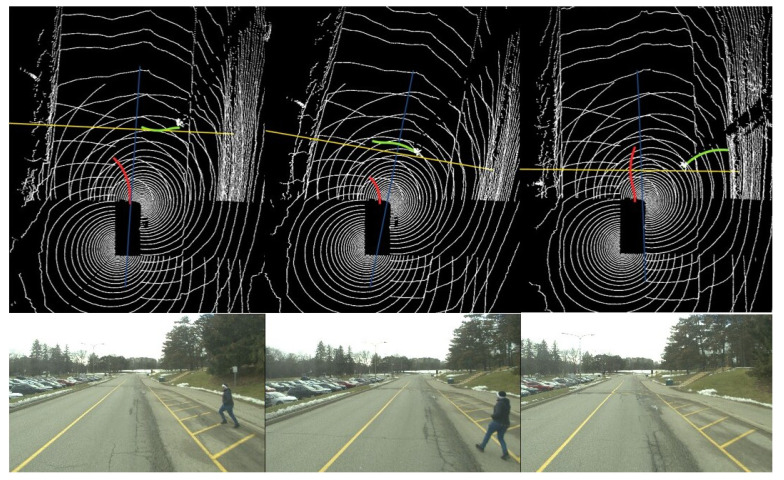
WATonoBus and pedestrian interaction using the Stackelberg motion planner (Red line: AV’s planned trajectory; Green line: pedestrian’s predicted trajectory; Yellow line: pedestrian’s expected path).

**Figure 14:**
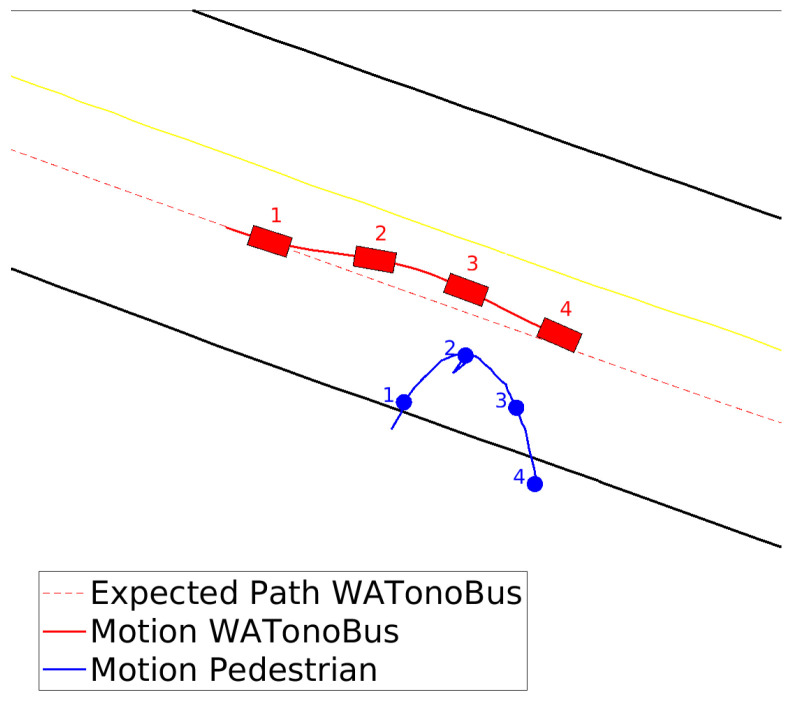
WATonoBus and pedestrian interaction, Stackelberg motion planner (Four distinct time steps are selected to show the locations of the pedestrian and the WATonoBus).

In the initial scenario, the Stackelberg motion planner is utilized to manage the interaction dynamics between the WATonoBus and a pedestrian. In this situation, the pedestrian suddenly attempts to cross the street, and given the pedestrian’s vulnerability, he is designated as the leader in this interaction. This approach enables us to model the interaction in a way that respects established traffic rules, positioning the pedestrian as the decision-maker who guides the initial moves within the game. The movement of both the WATonoBus and the pedestrian is illustrated in Figure 13. This figure displays the real-time performance of the motion planner in Rviz for three instances, including the planned trajectory for the AV (depicted as a red solid line) and the predicted trajectory for the pedestrian (depicted as a green solid line). The yellow solid line represents the expected path of the pedestrian using right and left curb information from the HD map, while the blue solid line shows the expected path of the AV. The white lines depicted in Figure 13 represent the LiDAR point clouds, providing a comprehensive spatial representation of the area scanned by the LiDAR system. Additionally, a camera view of this interaction is shown at the bottom of Figure 13. Notably, in the final scene of Figure 13, the pedestrian is out of range for the WATonoBus’s front camera, making him not visible in the corresponding image. However, due to the multilayer perception system, the pedestrian is still detected using other sensors, including side cameras, LiDAR, and RADAR. The WATonoBus initially deviates slightly from its expected path to the left and decreases its velocity as the length of the red path decreases in Figure 13. This adjustment shifts the AV’s position to the left of the expected path to reduce the risk of collision with the pedestrian. When the pedestrian senses the motion of the bus and returns to the crosswalk, the bus monitors this action. Subsequently, the Stackelberg motion planner redirects the AV back to its expected path and increases its velocity as the length of the red path increases. The entire interaction between the AV and the pedestrian is illustrated in Figure 14.

**Figure 15:**
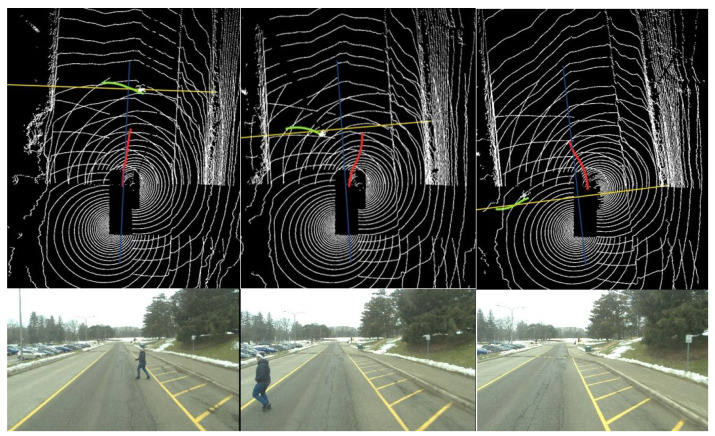
WATonoBus and pedestrian interaction using the Bayesian motion planner (Red line: AV’s planned trajectory; Green line: pedestrian’s predicted trajectory; Yellow line: pedestrian’s expected path).

**Figure 16:**
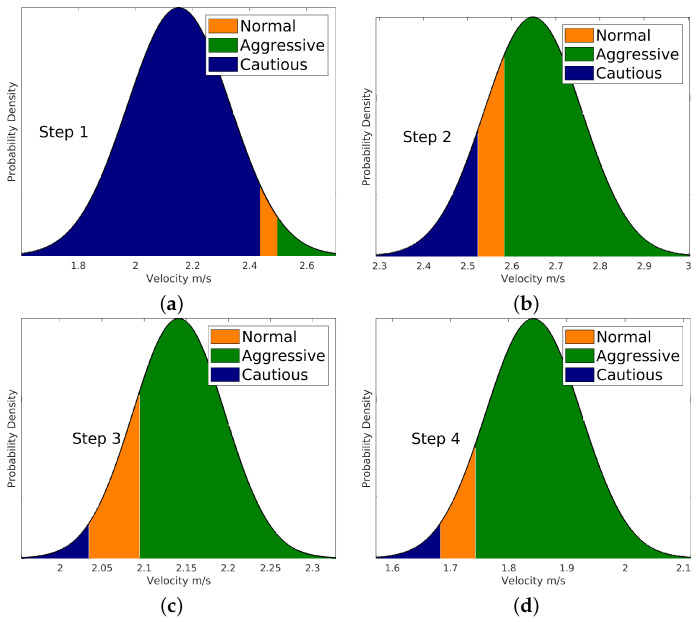
Probability distribution of pedestrian behavior. (**a**) First time step in the sequence. (**b**) Second time step in the sequence. (**c**) Third time step in the sequence. (**d**) Fourth time step in the sequence.

**Figure 17:**
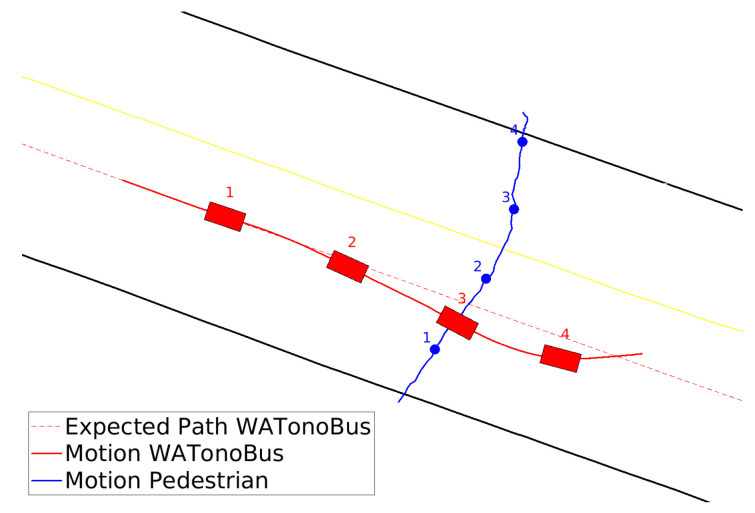
WATonoBus and pedestrian interaction, Bayesian motion planner (Four distinct time steps are selected to show the locations of the pedestrian and the WATonoBus).

In the second scenario, the WATonoBus navigates the Ring Road using the Bayesian motion planner. This planner enhances its motion-planning process by leveraging prior tracking data on pedestrian movement. By integrating Bayesian principles, it addresses the inherent uncertainty of sensor measurements. As a result, the Bayesian motion planner considers not only the current state of the pedestrian detected by sensors but also historical movement data to predict potential future actions. If there is an error in the current measurement of pedestrian velocity, comparing it with historical data helps mitigate this error. To accurately derive the pedestrian’s behavior, the velocities from the previous 10 time steps are recorded. The real-time interaction between the WATonoBus and the pedestrian is visually depicted in Figure 15. Additionally, the pedestrian’s probability distribution is illustrated in Figure 16 for selected instances. Similar to the previous scenario, in the final scene of Figure 15, the pedestrian is outside the front camera’s field of view, making him not visible in the last image. Initially, based on sensor measurements, the Bayesian motion planner classifies the pedestrian as a cautious road user, as seen in step 1. However, as time progresses and new measurements are acquired (steps 2, 3, and 4), the pedestrian is categorized as a more aggressive road user. At each time step, the WATonoBus continuously updates its belief probability function about the pedestrian by incorporating the collected and recorded velocity data from the pedestrian’s movements. This information influences how the AV reacts to the pedestrian’s actions. Initially, the WATonoBus moves to the right of its expected path, and once it passes the pedestrian, the Bayesian motion planner directs the AV back to its expected path. The complete interaction between the AV and the pedestrian, while the AV navigates using the Bayesian motion planner, is depicted in Figure 17.

When assessing the performance of the Bayesian and Stackelberg motion planners, it becomes evident that in the Bayesian motion planner, where both participants engage in a non-cooperative game without any hierarchical structure, the WATonoBus approaches the pedestrian much more closely than it does when utilizing the Stackelberg motion planner. Furthermore, when the AV utilizes the Bayesian motion planner, there are no significant fluctuations in its velocity since the path length remains constant. As a result, the probability function distribution primarily influences the direction of the AV’s planned path.

## 6. Conclusions

In current research, an innovative framework is presented, designed to tackle the complex challenges inherent in AV motion planning. This framework leverages the principles of both Stackelberg equilibrium and Bayesian equilibrium, enabling a comprehensive approach to address road traffic dynamics. In recognizing the hierarchical nature of interactions among road users, the proposed approach strategically optimizes the AV’s trajectory while considering the intentions and priorities of other entities using the Stackelberg game model, ensuring the AV’s compliance with traffic regulations.

The incorporation of Bayesian equilibrium adds a layer of adaptability to the AV’s motion-planning process, particularly in managing the uncertainty stemming from sensor measurements. By integrating probabilistic beliefs and continually updating them based on sensor data, the AV can make well-informed choices that take into account not only the immediate information at hand but also a historical perspective of collected data. The outcomes derived from the experimental assessments continue to highlight the promise of this framework. They emphasize the framework’s capacity to enhance the reliability and adaptability of AV motion planning, showing promise in real-world applications where dynamic traffic scenarios demand intelligent and adaptive responses.

Future work will focus on leveraging advanced intelligence techniques, including deep learning, reinforcement learning, and behavioral modeling, to gain a deeper understanding of road users’ behaviors and their initial intentions. Moreover, additional research will investigate the integration of real-time multimodal data from Vehicle-to-Vehicle (V2V) and Vehicle-to-Infrastructure (V2I) communication systems to enhance the AV’s comprehension of the road environment.

## Figures and Tables

**Table 1:** Bimatrix related to the Stackelberg game (Red cells indicate the best response of the AV to the leader’s actions).

	$p_{1}^{1}$	$p_{1}^{2}$	$p_{1}^{3}$	$p_{1}^{4}$	$p_{1}^{5}$	$p_{1}^{6}$
$p_{2}^{1}$	3.3∣8.4	6.6∣6.5	6.5∣3.1	6.6∣5.9	2.0∣5.7	0.2∣5.4
$p_{2}^{2}$	1.7∣1.3	7.9∣5.3	3.3∣4.7	7.9∣6.5	7.8∣1.0	6.8∣3.5
$p_{2}^{3}$	4.9∣0.6	8.6∣6.7	2.6∣8.4	9.0∣4.7	1.2∣4.1	3.3∣8.5
$p_{2}^{4}$	5.3∣3.8	5.3∣2.9	9.4∣3.2	4.8∣0.9	4.8∣8.0	9.6∣7.0
$p_{2}^{5}$	7.2∣1.0	8.6∣0.8	5.2∣8.6	3.7∣6.4	3.1∣0.5	1.2∣9.5
$p_{2}^{6}$	6.4∣4.1	5.1∣7.6	6.4∣4.9	3.8∣9.5	1.7∣9.0	6.4∣3.5

**Table 2:** Game specification for simulation.

Parameters	Value	Parameters	Value
δϕ	0.05 rad	δv	0.55 m/s
$K_{v}$	0.8	$K_{D}$	0.2
$K_{G}$	0.8	$K_{L}$	4
$K_{C}$	6	Prediction horizon	12
$s_{1}$	21	$s_{2}$	21

**Table 3:** Normal behavior bimatrix related to Bayesian game.

	$p_{1}^{1}$	$p_{1}^{2}$	$p_{1}^{3}$	$p_{1}^{4}$	$p_{1}^{5}$	$p_{1}^{6}$
$\rho_{2}^{1}\left(\theta_{2}^{n}\right)$	3.3∣8.4	6.6∣6.5	6.5∣3.1	6.6∣5.9	2.0∣5.7	0.2∣5.4
$\rho_{2}^{2}\left(\theta_{2}^{n}\right)$	5.3∣3.8	5.3∣2.9	9.4∣3.2	4.8∣0.9	4.8∣8.0	9.6∣7.0

**Table 4:** Aggressive behavior bimatrix related to Bayesian game.

	$p_{1}^{1}$	$p_{1}^{2}$	$p_{1}^{3}$	$p_{1}^{4}$	$p_{1}^{5}$	$p_{1}^{6}$
$\rho_{2}^{1}\left(\theta_{2}^{a}\right)$	1.7∣1.3	7.9∣5.3	3.3∣4.7	7.9∣6.5	7.8∣1.0	6.8∣3.5
$\rho_{2}^{2}\left(\theta_{2}^{a}\right)$	7.2∣1.0	8.6∣0.8	5.2∣8.6	3.7∣6.4	3.1∣0.5	1.2∣9.5

**Table 5:** Cautious behavior bimatrix related to Bayesian game.

	$p_{1}^{1}$	$p_{1}^{2}$	$p_{1}^{3}$	$p_{1}^{4}$	$p_{1}^{5}$	$p_{1}^{6}$
$\rho_{2}^{1}\left(\theta_{2}^{c}\right)$	4.9∣0.6	8.6∣6.7	2.6∣8.4	9.0∣4.7	1.2∣4.1	3.3∣8.5
$\rho_{2}^{2}\left(\theta_{2}^{c}\right)$	6.4∣4.1	5.1∣7.6	6.4∣4.9	3.8∣9.5	1.7∣9.0	6.4∣3.5

**Table 6:** Expanded bimatrix Bayesian game (The red cell indicates the equilibrium point in the bimatrix Bayesian game).

	$p_{1}^{1}$	$p_{1}^{2}$	$p_{1}^{3}$	$p_{1}^{4}$	$p_{1}^{5}$	$p_{1}^{6}$
$\rho_{2}^{1}\left(\theta_{2}^{n}\right)\rho_{2}^{1}\left(\theta_{2}^{a}\right)\rho_{2}^{1}\left(\theta_{2}^{c}\right)$	3.8∣3.5	7.8∣6.4	4.1∣5.9	7.9∣5.4	2.6∣4.1	2.8∣6.5
$\rho_{2}^{1}\left(\theta_{2}^{n}\right)\rho_{2}^{1}\left(\theta_{2}^{a}\right)\rho_{2}^{2}\left(\theta_{2}^{c}\right)$	4.5∣5.2	6.1∣6.8	5.9∣4.2	5.5∣7.7	2.8∣6.5	4.2∣4.2
$\rho_{2}^{1}\left(\theta_{2}^{n}\right)\rho_{2}^{2}\left(\theta_{2}^{a}\right)\rho_{2}^{1}\left(\theta_{2}^{c}\right)$	4.7∣3.5	7.9∣5.6	4.4∣6.5	7.2∣5.4	1.8∣4.1	1.8∣7.6
$\rho_{2}^{1}\left(\theta_{2}^{n}\right)\rho_{2}^{2}\left(\theta_{2}^{a}\right)\rho_{2}^{2}\left(\theta_{2}^{c}\right)$	5.4∣5.1	6.2∣6.0	6.2∣4.9	4.8∣7.7	2.0∣6.4	3.3∣5.2
$\rho_{2}^{2}\left(\theta_{2}^{n}\right)\rho_{2}^{1}\left(\theta_{2}^{a}\right)\rho_{2}^{1}\left(\theta_{2}^{c}\right)$	4.5∣1.9	7.3∣5.1	5.2∣5.9	7.3∣3.6	3.6∣5.0	6.2∣7.1
$\rho_{2}^{2}\left(\theta_{2}^{n}\right)\rho_{2}^{1}\left(\theta_{2}^{a}\right)\rho_{2}^{2}\left(\theta_{2}^{c}\right)$	5.2∣3.5	5.6∣5.5	7.0∣4.3	4.9∣5.9	3.9∣7.3	7.6∣4.8
$\rho_{2}^{2}\left(\theta_{2}^{n}\right)\rho_{2}^{2}\left(\theta_{2}^{a}\right)\rho_{2}^{1}\left(\theta_{2}^{c}\right)$	5.4∣1.8	7.4∣4.3	5.5∣6.6	6.6∣3.6	2.8∣4.9	5.2∣8.1
$\rho_{2}^{2}\left(\theta_{2}^{n}\right)\rho_{2}^{2}\left(\theta_{2}^{a}\right)\rho_{2}^{2}\left(\theta_{2}^{c}\right)$	6.1∣3.5	5.8∣4.8	7.3∣4.9	4.1∣5.9	3.1∣7.2	6.7∣5.8

**Table 7:** Performance of motion planners in the overtaking scenario.

Planner Type	ILQ	Stackelberg	Bayesian (Aggressive Case)	Bayesian (Cautious Case)
Calculation time (s)	6.14	1.05	1.50	1.45

## Data Availability

Data are contained within the article.
